# Paternal Lineage and Genetic Diversity of Jiuzhi Yaks Revealed by Y-Chromosome SRY Sequencing

**DOI:** 10.3390/ani15192783

**Published:** 2025-09-24

**Authors:** Boxuan Yang, Xiaolin Luo, Tianwu An, Jiuqiang Guan, Xiangfei Zhang, Qin Bai, Quan Sha, Hongwen Zhao

**Affiliations:** Sichuan Academy of Grassland Sciences, Chengdu 611731, China; ybx422830@163.com (B.Y.); luoxl2004@sina.com (X.L.); antianwu@126.com (T.A.); gjq1900@163.com (J.G.); zxfsicau@foxmail.com (X.Z.); bq1278560523@163.com (Q.B.); 17360296361@163.com (Q.S.)

**Keywords:** yak, patrilineal inheritance, SRY gene, genetic evolution

## Abstract

Yak husbandry on the Qinghai–Tibetan Plateau relies on genetically resilient sire lines, yet the paternal ancestry of the locally prominent Jiuzhi breed has never been quantified. To resolve this gap, a 690 bp fragment of the Y chromosomal SRY gene was sequenced in 117 males spanning Jiuzhi (n = 12) and five neighboring Qinghai breeds and compared with three single individual public records that represent Qinghai Plateau, Tianzhu White, and Wild yaks. Alignment, haplotype calling, diversity indices, F_ST differentiation, AMOVA, multidimensional scaling, and TCS network analysis were performed. Thirty-two haplotypes were recovered; Jiuzhi yaks possessed twelve, and six of them had private alleles. Mean A + T content was 53.8%. Haplotype diversity was highest in Qinghai Plateau, Tianzhu White and Wild yaks and lowest in Larima yaks, while Jiuzhi diversity resembled that of Huanhu. Pairwise F_ST values indicated negligible differentiation between Jiuzhi and either Qinghai Plateau or Yushu yaks (F_ST ≈ 0) but pronounced divergence from Tianzhu White and Wild yaks (F_ST ≈ 0.97–0.99). AMOVA attributed 90.7% of molecular variance to among-breed differences, and multidimensional scaling clustered Jiuzhi with Jinchuan, Yushu and Huanhu. Network topology resolved two patrilineal clades, confirming dual paternal origins for Jiuzhi yaks. These data provide the first quantitative baseline for sire selection and conservation of Jiuzhi yaks and highlight close affinities with adjacent plateau breeds. Limitations include the use of single individual public sequences for Qinghai Plateau, Tianzhu White and Wild yaks (employed solely as phylogenetic anchors) and reliance on a single Y-linked locus; broader sampling and multilocus approaches will refine within-breed estimates.

## Simple Summary

Accurate knowledge of male lineages helps herders select bulls that keep unique traits while boosting herd productivity. We sequenced the SRY gene in 117 male yaks from eight breeds and found that Jiuzhi yaks derive from two paternal lineages with moderate diversity. Breeds such as Tianzhu White and Wild yaks are genetically distant, so that uncontrolled crossbreeding could erode rare alleles. Our results guide producers in choosing sires that safeguard local genes and improve performance under plateau conditions.

## 1. Introduction

Patrilineal inheritance is the transmission of genetic information to offspring through the father. Unlike matrilineal inheritance, paternal inheritance is mainly transmitted through the Y chromosome or some specific genetic markers. The Y chromosome in mammals is essential for male sexual development, spermatogenesis, and male fertility [1]. The Y chromosome is paternally inherited, exhibits a low mutation rate and strong haplotype integrity, which makes it an important means of studying paternal genetic diversity, domestication history, and evaluating the evolution of its origins [2]. The mammalian Y chromosome occurs as a single copy in males and is the smallest chromosome, harboring the fewest protein-coding genes. Therefore, Y-linked loci are predominantly haploid, and the Y chromosome contains approximately 60 genes that are expressed exclusively in the testes [3]. Some scholars believe that the X and Y chromosomes originated from autosomes, but through evolution and natural selection, one of the chromosomes gradually degenerated, resulting in the inactivation of specific genes [4]. It has also been found that a new sex-determining gene, SRY, exists on the X chromosome with the SXO3 mutation. The mutation leads to the splitting of the X and Y chromosomes, followed by a series of differentiation and evolution, which ultimately gives rise to the Y chromosome. This chromosome plays a vital regulatory role in male reproduction in mammals [5]. SRY is a gene that plays a crucial role in the Y chromosome, promoting testicular differentiation and suppressing ovarian development [6]. The SRY gene is the first SOX family member to be identified, and it plays a crucial role in maintaining the number of stem cells, regulating apoptosis, and promoting cell differentiation [7]. In recent years, the study of SRY genes in yak (Bos grunniens) has been important for understanding sex determination mechanisms, genetic diversity, and evolution. Some progress has been made in the study of SRY gene in yak, Zhang et al. analyzed SRY gene in 24 domestic and foreign sheep germplasm resources, and the results showed that gene gradual infiltration occurred in both Xinjiang Hetian and Ningxia Tangshan sheep [8]. Liang cloned the SRY genes of forest musk and horse musk, and found that the full length of the SRY genes of forest musk and horse musk was 684 bp, which contained 227 amino acids in the HMG-box region [9]. The current research on the SRY gene of horse musk is still limited, and it was found in GeneBank database that the full length of horse musk SRY gene was 690 bp; the above studies covered gene sequence analysis, polymorphism study, sex determination mechanism, evolutionary analysis and application development, etc. Future studies will further elucidate the function and regulatory mechanism of the SRY gene, thereby providing scientific support for the genetic improvement and breeding management of yaks.

The Jiuzhi yak is a genetically distinct, indigenous breed inhabiting the Qinghai–Tibet Plateau, primarily distributed in Jiuzhi County within the Guoluo Tibetan Autonomous Prefecture of Qinghai Province, and in adjacent high-altitude pastoral regions. It is an excellent livestock species adapted to the extreme environment of the plateau, formed by Tibetan herders through long-term natural selection and artificial domestication, and it is one of the most important means of production and living, as well as an important source of income for the Tibetan population in the pastoral areas of Jiuzhi. Little information exists on the paternal lineage of Jiuzhi yaks; previous work mainly relied on mitochondrial or autosomal markers. By sequencing a 690 bp segment of SRY and comparing it with neighboring breeds, we fill this gap and provide the first Y-chromosome perspective on the Jiuzhi lineage. We hypothesized that geographic isolation has produced Jiuzhi-specific Y-chromosome haplotypes distinct from adjacent yak populations. Therefore, the paternity genetic analysis of Jiuzhi yaks has important theoretical and practical values, especially in the identification of breed resources, genetic diversity conservation, and optimization of breeding strategies. In current study, a comparative paternal genetic analysis of the Jiuzhi yak and other yak populations based on polymorphisms within the sex-determining region Y (SRY) of the Y chromosome was conducted. This analysis aims to generate foundational data to inform genetic improvement programs and guide breeding management practices for this locally valuable yak breed.

## 2. Materials and Methods

### 2.1. Animal Sample Collection

In Jiuzhi County, Guoluo Prefecture, Qinghai Province, 12 Jiuzhi male yaks were selected, blood was collected from the jugular vein, placed in a sample box for low temperature storage, and sent for testing with blood samples, as well as sequences of eight yak breed populations of 117 male yaks downloaded from the GenBank database (1 Highland Yak, 1 Tianzhu White Yak, and 1 Wild Yak), and compared and clipped for homology to the sequences of the measured breed yak populations. A total of 117 male yaks from 8 yak breed groups (1 plateau yak, 1 Tianzhu white yak, and one wild yak) downloaded from the GenBank database were sequenced and clipped to a homologous 690 bp with the sequences of the measured breed yak groups to facilitate the subsequent data analysis. Because each of the Qinghai Plateau, Tianzhu White, and Wild yak breeds is represented by a single public sequence, these records function only as phylogenetic anchors. They are omitted from diversity, differentiation, and AMOVA statistics that require multiple individuals (Table 1 and Table 2).

### 2.2. Main Reagents and Instruments

#### Main Reagents

Taq Plus DNA Polymerase, 10_X_ PCR Buffer (with Mg^2+^), dNTP (10 mM), Sterilized deionized water, primer DNA, EDTA, NaAc, NaOH, all are derived from (bioengineering), 6_X_ DNA Loading Dye root in (Thermo Fisher, Waltham, MA, USA) (Supplementary Table S1).

### 2.3. Genomic DNA Extraction

Genomic DNA was isolated from 200 µL whole blood aliquots with the Ezup Column Animal Tissue Genomic DNA Extraction Kit (Tiangen Biotech, Beijing, China; Lot EK-0512), which incorporates silica membrane binding and on-column RNase digestion. Eluates (50 µL) were quality screened in two steps. First, 5 µL of each sample was electrophoresed on a 1% agarose gel in 1× TAE at 150 V for 25 min; intact, high molecular weight bands without smearing confirmed that genomic DNA was undegraded and present at amplifiable levels. Second, nucleic acid concentration and purity were assessed on a Merinton SMA4000 UV-Vis spectrophotometer (Merinton Instruments Ltd. Shanghai, China): samples with A_260_/A_280_ ratios between 1.7 and 2.0 were deemed protein free, whereas values < 1.7 or >2.0 indicated protein or RNA contamination, respectively. DNA passing both checks was adjusted with nuclease free water to 50–100 ng µL^−1^ and stored at −20 °C until PCR amplification.

### 2.4. Primer Design, PCR Amplification, and Sequence Determination

PCR amplification was performed by targeting the SRY4 Y-SNP marker described in a previous study [10]. Primer quality was first verified for hairpins, dimers, and ΔG values in OligoAnalyzer v3.1 (Integrated DNA Technologies, Coralville, IA, USA). The validated oligonucleotides forward 5′-GTCTGCTGCACCTTCATC-3′ and reverse 5′-CTTATTGTGGCCCAGGCTTGTC-3′ span 969 bp and were synthesized by Sangon Bioengineering (Shanghai, China). Each 25 µL reaction contained 1× PCR buffer with Mg^2+^, 200 µM of each dNTP, 0.4 µM of each primer, 1 U of Taq Plus DNA polymerase v2.0 (Tiangen, Beijing, China), and ~50 ng of template DNA. Thermal cycling was performed on an ABI Veriti 96 well thermocycler under the following regime: initial denaturation at 95 °C for 3 min; 35 cycles of 95 °C for 30 s, 62 °C for 45 s and 72 °C for 60 s; and a final extension at 72 °C for 10 min. Amplicons were visualized on 1% agarose gels in 1× TAE, excised, purified with a Gel-X kit (Tiangen, Beijing, China), and submitted to Sangon Bioengineering for bidirectional Sanger sequencing on an ABI 3730XL platform.

### 2.5. Data Analysis

Raw chromatograms were inspected for signal quality, trimmed to a uniform 690 bp, and exported in FASTA format using Chromas Lite v2.6.6. High-accuracy multiple alignment of all sequences was then carried out with ClustalW v2.1, and the resulting consensus block was converted to NEXUS format in DnaSP v6.0 [11] to facilitate downstream population analyses. From this alignment, we generated an ARP file that was processed in Arlequin v3.11 [12], yielding standard diversity metrics and an analysis of molecular variance (AMOVA). To visualize interpopulation structure, pairwise F_ST values were linearized and subjected to two-dimensional multidimensional scaling in IBM SPSS Statistics v27.0. Phylogenetic relationships among haplotypes were reconstructed with the Tamura Nei model in PAUP v4.0, generating both neighbor joining and UPGMA dendrograms from the same NEXUS dataset. Finally, population-specific haplotype counts were appended to the NEXUS file, and a statistical parsimony (TCS) network was inferred in PopART v1.7 [13], providing an intuitive visual summary of patrilineal connectivity across breeds.

## 3. Results

### 3.1. The Frequency Distribution Among the Eight Yak Breeds (Populations)

**Figure 1:**
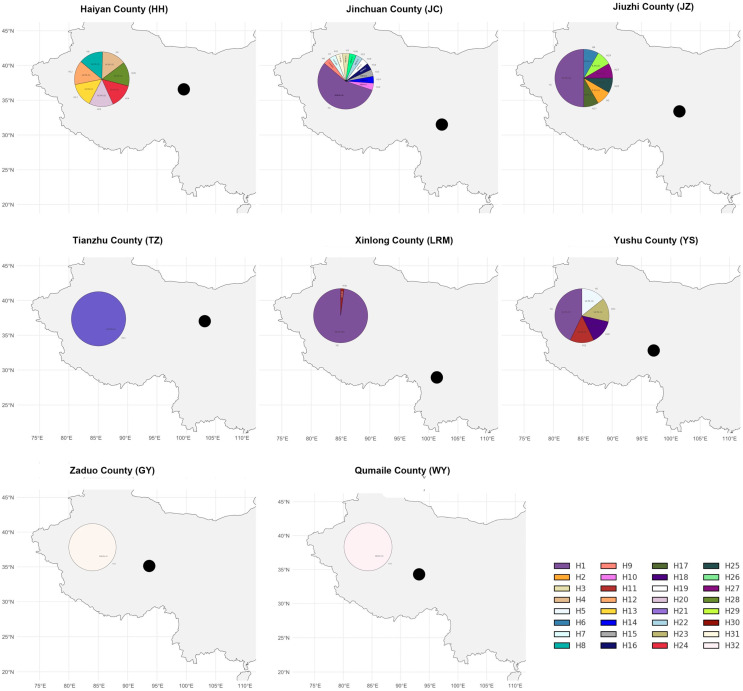
Spatial distribution of 32 Y-chromosome SRY haplotypes in eight Qinghai yak populations. Pie charts are centered on sampling counties (HH = Haiyan, JC = Jinchuan, JZ = Jiuzhi, TZ = Tianzhu, LRM = Larima/Xinlong, YS = Yushu, GY = Zaduo, WY = Qumalai). Slice area is proportional to haplotype frequency; colors match the legend (H1–H32). Single-color pies (TZ, GY, WY) indicate populations represented by one public reference sequence only. The black dots show the location of each county in the region.

According to our findings, only a few yak breeds (populations) share precisely the same haplotype composition, while most breeds (populations) have specific differences in haplotype composition. Among all the breeds (populations), only Tianzhu White Yak and Qinghai Plateau Yak have the same haplotype composition. Both Tianzhu White Yak and Qinghai Plateau Yak have only one haplotype, namely H21. In addition, the haplotype compositions of other yak breeds (populations) are not entirely the same. Additionally, the frequencies of different haplotypes within each yak breed (population) also vary. For example, in the Huanhu Yak, seven haplotypes were detected, namely H4, H8, H12, H13, H20, H24, and H28, all occurring at similar frequencies; in the Jinchuan Yak, twelve haplotypes were detected, namely H1, H3, H7, H9, H10, H14, H15, H16, H19, H22, H26, and H31, with H1 having the highest frequency and the rest having consistent frequencies; Jiuzhi Yak has H1, H2, H6, H17, H25, H27, and H29, with H1 having the highest frequency; Larima Yak has two haplotypes, namely H1 and H30, with H1 having the highest frequency; Yushu Yak has five haplotypes, namely H1, H5, H11, H18, and H23, with H1 having the highest frequency followed closely by H5, while the remaining three show similar frequencies (Figure 1).

### 3.2. Base Composition of SRY Gene Fragments of Yak Y Chromosome

An analysis of the length and base composition of Yak Y chromosome SRY gene fragments revealed that the fragments of 8 yak populations were 690 bp. Additionally, the nucleotide composition of the SRY gene fragments was analyzed in these populations. The contents of 4 nucleotides, as well as A + T% and G + C% of each population, are shown in Table 3. The average contents of the four bases, A, T, C, and G, were 30.03%, 21.47%, 24.73%, and 23.77%, respectively, in the 17 yak populations’ SRY gene fragments. C, G, and T% were 30.03%, 21.47%, and 24.73%, respectively, with A + T% and G + C% totaling 53.80% and 46.19%, respectively. The higher A + T content compared to G + C content indicated that the SRY gene region of yak was enriched in A + T. The lengths of eight yak populations were consistent among individuals and breeds. The length of the SRY gene region in the eight yak populations was consistent among individuals and breeds, suggesting that it does not affect gene expression regulation, nor does it exerts selection pressure on evolutionary adaptations. The high G + C content may be related to gene stability, antiretroviral insertion, or transcriptional regulation.

### 3.3. Haplotype Diversity Analysis of the SRY Region of the Yak Y Chromosome

The genetic diversity indices of the SRY region of the Y chromosome were analyzed and compared for a total of 117 individuals from eight yak populations. The results showed that a total of 32 haplotypes were defined in individuals of eight yak populations, among which the Jinchuan yak had the highest number of haplotypes with 12, the Huanhu yak had 10, and the Jiuzhi yak had 7 in the third place, and the above three yak populations had a higher number of haplotypes than the other populations; the haplotype diversity of the Plateau yak, the Tianzhu white yak, and the wild yak (1.000 ± 0.000) was much higher than that of the other yak populations, of which the Jiuzhi yak and the Jiuzhi yak had the third largest haplotype diversity. Was much higher than that of other yak groups, among which the haplotype diversity of Jiuzhi yak and Huanhu yak did not differ much, with (0.773 ± 0.128) and (0.711 ± 0.114), respectively; the smallest index of haplotype diversity was found in the Lajima yak (0.040 ± 0.038); the nucleotide diversity of Jiuzhi yak was the same as that of the Huanhu yak and Yushu yak (0.003 ± 0.002) The average nucleotide diversity of Jiuzhi yak and Yushu yak was (2.000 ± 1.211) and (2.000 ± 1.276), respectively, which was higher than that of the other yak groups (Table 4).

### 3.4. Analysis of Paternal Genetic Differentiation Among Yak Breeds

By analyzing the intergroup F_ST values in the SRY region of the Y chromosome in yak populations, the degree of paternal genetic differentiation among different yak populations and its potential drivers can be assessed using the fixed differentiation index (F_ST) values among eight yak populations (Table 5). The results showed that the eight yak populations, with F_ST values ranging from −0.001 to 1, exhibited different degrees of significant genetic differentiation (*p* < 0.05). The F_ST values of the Jiuzhi yak population, the plateau yak, and the Yushu yak were 0, indicating frequent gene exchange between the populations and low genetic differentiation. The F_ST values of the Jiuzhi yak population, the Tianzhu white yak, and the wild yak were 0.968 and 0.994, indicating that the populations were highly isolated from each other and that the flow of genes was limited. The average F(ST) values of plateau yaks, Tianzhu white yaks, and wild yaks were the highest (0.915), while those of Jiuzhi yaks were the lowest (0.900), suggesting that Jiuzhi yaks are more genetically homogeneous than several other yak populations.

### 3.5. Multidimensional Scaling Analysis Among Yak Breeds

As shown in the MDS plot (Figure 1), the eight yak breeds (groups) could be obviously clustered into four classes in the PC1-PC2 two-dimensional coordinate combination, among which the Wild Yak (WY) and Tianzhu White Yak (TZ) were clearly separated from the other yak breeds (groups) as their coordinate distances were farther away from each other in the plot, and the two of them were farther away from each other as well, so each of them was classified into one class; the Larima Yak (LRM) and Qinghai plateau yak (GY) are closer to each other and clustered into one category; Jinchuan yak (JC) and Jiuzhi yak (JZ), Yushu yak (YS), and Huanhu yak (HH), the inter-breeds (groups) are closer to each other and clustered into one category. It is implied that these yak breeds (populations) have similar paternal genetic components and are clustered into another category.

### 3.6. Analysis of Molecular Variation in Yak

Analysis of molecular variation (AMOVA) of Y-chromosome haplotypes in domestic yaks showed (Table 6) that 90.71% of the variation was distributed within yak breeds (populations), and 9.29% of the variation components were distributed among breeds (populations) within groups. The summed genetic variation among breeds (populations) as well as within breeds (populations) within yak groups reached a significant level (*p* < 0.0001), indicating that genetic variation among breeds (populations) as well as within breeds (populations) of yak has a greater degree of influence on their genetic structure. The high percentage of inter-population we hypothesized could be due to geographic isolation or intense selection pressure leading to differentiation.

### 3.7. Phylogenetic Network Diagram Analysis of Y Chromosome Haplotypes in Six Yak Breeds (Populations)

**Figure 2:**
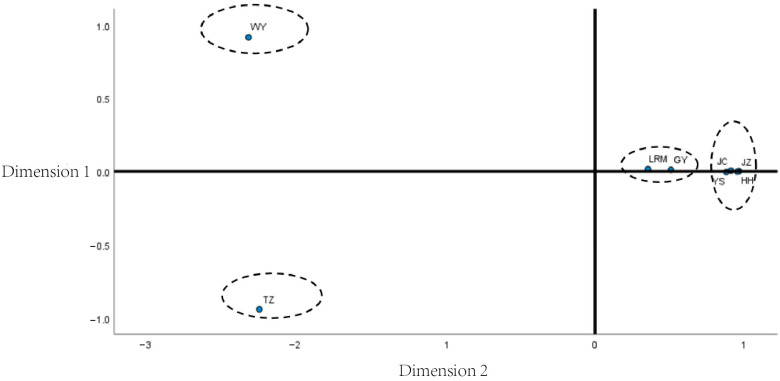
Multidimensional scaling (MDS) plot derived from pairwise F_ST values among eight yak populations. Points represent population centroids; 95% confidence ellipses illustrate dispersion. The configuration resolves four clearly separated clusters, with Wild (WY) and Tianzhu White (TZ) forming the most divergent group.

**Figure 3:**
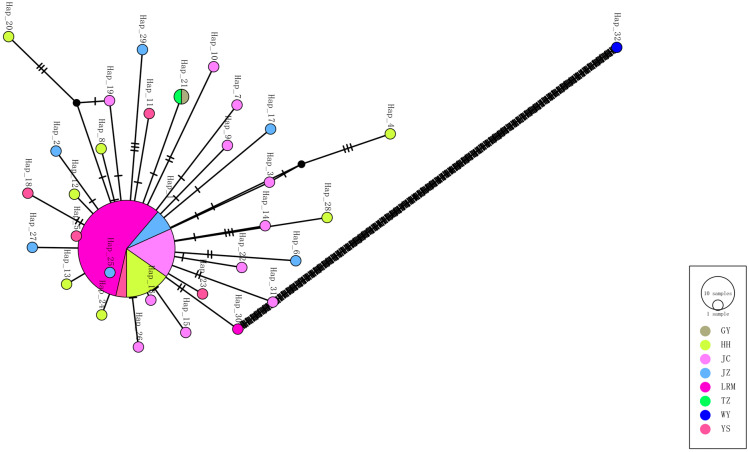
TCS parsimony network of 32 SRY haplotypes. Circle diameter is proportional to haplotype frequency; segment colors identify populations (legend). Hatch marks denote single mutational steps. Two patrilineal clades are evident: a star-like Y1 cluster containing 31 haplotypes and an isolated Y2 lineage represented solely by the wild yak sequence. The Dash circles represent median vectors, and the bold line indicates a long mutational branch, with each dash corresponding to a single nucleotide substitution.

**Figure 4:**
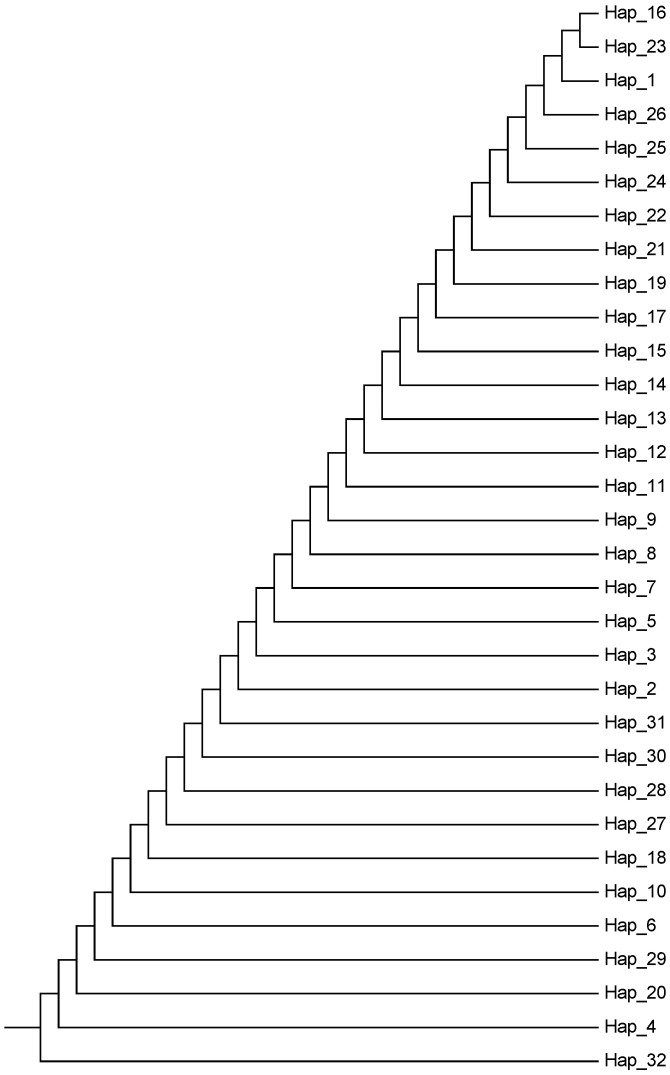
UPGMA dendrogram of SRY haplotypes calculated with Tamura-Nei distances. Bootstrap values greater than 50% from 1000 pseudo-replicates are shown at nodes. The topology corroborates the Y1/Y2 bifurcation recovered in the TCS network.

The results of Y chromosome haplotype network analysis (Figure 2) showed that the 32 haplotypes identified could be divided into two distinct haplotype groups, Y1 and Y2, of which the Y1 haplotype group consisted of 31 haplotypes ranging from H1Y1 to H31Y1, while the Y2 haplotype group consisted of only one haplotype, H32Y2, which suggests that yaks consist of two patrilineal clades. A total of 12 haplotypes were generated in the Jiuzhi yak group, of which six were endemic. The Jiuzhi yak group shared H1Y1 with the Huanhu yak, Jinchuan yak, Larima yak, and Yushu yak groups, while H2Y1, H6Y1, H17Y1, H25Y1, H27Y1, and H29Y1 were exclusively found in the Jiuzhi yak group. We found that the Jiuzhi yak group shared fewer haplotypes with other yak breeds. We hypothesized that the Jiuzhi yaks had been living in a specific geographical environment for a considerable amount of time. Their distribution areas were far away from those of other yak breeds, which prompted them to evolve specific genes adapted to the local environment. The haplotypes formed by the combinations of these genes were more different from those of other yak breeds with different growing environments, and thus, fewer haplotypes were shared. Overall, yaks were dominated by the Y1 haplotype group/clade and supplemented by the Y2 haplotype group/clade, with the Y1 haplotype group/clade owning 99.1% (115/116) of the yak individuals, while the Y2 haplotype group/clade owned only 0.90% (1/116) of the total yak heads. In addition, the network relationship diagram showed that haplotype H25Y1 was at the core of the Y1 haplotype group/clade, with other haplotypes dispersed around it in a star-like pattern. Notably, haplotype H32Y2 was only present in the wild yak population. Therefore, it is hypothesized that haplotypes H25Y1 and H32Y2 are the two oldest core haplotypes in yaks and represent the most primitive haplotypes in the two yak clades. Refer to Figure 3, which, using the haplotype clustering UPGMA plot results, again verifies that the haplotype network plot analysis was relevant. This bifurcation was further confirmed by the UPGMA dendrogram of SRY haplotypes, which clearly grouped the haplotypes into two distinct clades (Figure 4).

## 4. Discussion

Genetic diversity refers to the variation in genetic composition within and between biological populations, primarily characterized by differences in allele frequencies among individuals and populations. It serves as a fundamental component of biodiversity, influencing evolutionary potential, population adaptability, and species resilience to environmental changes [14]. As an important driver of species evolution, its abundance directly affects the ecological adaptability, survival competitiveness, and genetic evolution potential of species. In the livestock production system, the level of genetic diversity has a significant correlation with the sustainable utilization of livestock germplasm resources [15]: when the heterogeneity of the gene pool is below the critical threshold, it will trigger the inbreeding decline phenomenon, which is manifested in the reduction in reproductive efficiency, weakening of disease resistance and the decline of production performance, etc.; and a high level of genetic diversity provides abundant genetic material for breed improvement, which is conducive to the construction of a multi-dimension selection index, and thus enhances the genetic evolution potential of livestock. The high level of genetic diversity provides rich genetic material for breed improvement, which is conducive to the construction of a multi-dimensional selection index. It thus enhances the buffering capacity of the herd against environmental stresses and the group’s resistance to epidemics. Such polymorphic characterization can provide a quantitative basis for assessing the status of germplasm resource reserves, which is a significant reference value for developing scientific genetic material conservation strategies.

Previous researchers have also analyzed the genetic diversity of the Y chromosome in yaks across different yak breeds. A study examined a total of 322 yak individuals from three yak populations, namely the plateau yak, the Huanhu yak, and the Datong yak, based on 10 Y-SNPs, and identified seven Y chromosome haplotypes, and the haplotype clustering and network analyses deduced that there were two haplogroups, Y1 and Y2, which indicated that there were two different lineages in the paternal origins of the yak in Qinghai [16]. The Y chromosome haplotype diversity was 0.538 ± 0.028, indicating a high level of diversity in the Qinghai yak population. Haplotype diversity was similar in plateau yak and Datong yak breeds (0.547 ± 0.030 and 0.553 ± 0.083, respectively), and higher than that of the Huanhu breeds (0.441 ± 0.098). Genetic diversity analysis revealed that the four yak populations exhibited high paternal genetic diversity (Hd = 0.506), with the highest haplotype diversity observed in Mado yaks (Hd = 0.632) and the lowest in Gande yaks (Hd = 0.324). The results of this study, which included 117 male yaks from 8 yak groups, showed that a total of 32 haplotypes were defined. The haplotype diversity of plateau yaks, Tianzhu white yaks, and wild yaks was much higher than that of the other yak groups, with the haplotype diversity of Jiuzhi yaks and Huanhu yaks not differing much, with the haplotype diversity of (0.773 ± 0.128) and (0.711 ± 0.114), respectively; The haplotype diversity index of Larima yaks was the smallest (0.040 ± 0.038), which also verified the previous study and showed that yaks possessed relatively rich paternal genetic diversity.

Genetic variation within the mammalian Y chromosome provides critical insights into paternal lineage and male-mediated genetic diversity. Owing to the absence of recombination across most of its length, the male-specific region of the Y chromosome represents the most extended contiguous haplotype that is stably inherited across generations. In many species, reduced effective population sizes of males further amplify the phylogenetic signal of Y-linked markers, making them particularly valuable for tracing paternal ancestry. Consequently, Y chromosome variation often exhibits greater phylogeographic structure compared to mitochondrial DNA or autosomal loci [17,18,19].

F_ST measures the degree of genetic differentiation between varieties, with high F_ST values indicating large genetic distances [20]. According to Wright’s criteria for determining genetic differentiation between populations based on F_ST values [21]. If the F_STvalue is less than 0.05, it indicates that the genetic differentiation between populations is weak; if the F_STvalue is between 0.05 and 0.15, then the genetic differentiation between populations has reached an intermediate level; when the F_STvalue is between 0.15 and 0.25, then it indicates that there is a large degree of genetic differentiation between populations; when the F_STvalue is greater than 0.25, then the degree of differentiation between populations is When the F_ST value is greater than 0.25, the degree of differentiation between populations is significant. In this study, the Jiuzhi yaks were moderately genetically differentiated from the plateau yak, the lake yak, the Jinchuan yak, and the Yushu yak, more genetically differentiated from the Larima yak, and extremely genetically differentiated from the Tianzhu white yak and the wild yak. From the point of view of average F_ST values, the average F_ST values of Jiuzhi yaks were relatively low compared with other yak populations, and we hypothesized that the genetic homogeneity of Jiuzhi yaks was high compared with other yak populations. This suggests that there is a problem of deep differentiation in the Jiuzhi yak population. Subsequently, based on the F_ST values, MDS analysis was carried out. The results showed that the eight yak breeds (populations) could be obviously clustered into four classes, among which the Jiuzhi yak (JZ) and Jinchuan yak (JC), Yushu yak (YS), and Huanhu yak (HH), with closer coordinate distances between breeds (populations) were clustered into one class. It implies that Jiuzhi yaks have similar paternal genetic components to these yak breeds (populations) and are most closely related to them. The AMOVA analysis revealed that yaks primarily originated from inter-population exchange, and their intraspecific variation accounted for a smaller proportion. This suggests that there is significant genetic differentiation among different geographic populations of yaks, which may be related to long-term geographic isolation (e.g., blocked by the plateau mountain range), artificial selection pressures (directed selection for specific traits), or mating regimes (such as regional captive breeding). This result is consistent with the “islandized” distribution of yak populations in the special ecosystem of the Tibetan Plateau. The low proportion of intraspecific variation in the population suggests that the genetic base of the core population is narrowing, possibly due to historical bottleneck effects (e.g., population shrinkage resulting from climate extremes) or persistent inbreeding (repetition of a few highly productive bulls in a closed population). This phenomenon would accelerate the purification of deleterious recessive genes and reduce the population’s selective resilience to disease outbreaks or climate change.

As one of the core phylogenetic studies, the analysis of genetic structure of germplasm resources plays a key role in inverting the domestication history and genetic relatedness of species. The Y chromosome, due to its patrilineal genetic properties, offers a unique perspective for analyzing the paternal origin of yaks, sex-biased dispersal during domestication, and population history dynamics. Although the Y chromosome is generally less genetically diverse than mitochondrial DNA (mtDNA), its haplotype distribution and phylogenetic structure can reveal male-dominated gene flow and domestication events [22,23,24].

Studies on the mechanism of yak germplasm formation have shown that genealogical tracing based on Y chromosome-specific microsatellite markers (Y-STR) indicates the existence of two patrilineal haplogroups (Y1/Y2) with significant genetic distances in extant yak populations [25,26]. It is noteworthy that these two haplogroups exhibit a pan-geographic distribution across different ecological regions of the Tibetan Plateau, and do not form a pattern of genetic segregation associated with geomorphic units. It is hypothesized that the yak domestication process may have experienced a dual patrilineal origin event, and its formation mechanism may be related to the trans-regional migration of prehistoric human activities. In this study, a total of 117 male yaks from eight yak breed groups were systematically analyzed, and 32 haplotypes were identified, among which 12 haplotypes were found in Jiuzhi yaks, of which six were endemic. Jiuzhi yaks shared fewer haplotypes with other yak breeds, leading us to hypothesize that this breed has been living in a specific geographic area for an extended period. This isolation, combined with the breed’s geographical distance from other yak breeds, has contributed to the development of particular genes adapted to the local environment. Therefore, we hypothesize that this breed of yak has been living in a specific geographical environment for a long time and is geographically distant from other yak breeds, which has led to the evolution of particular genes adapted to the local environment. The haplotype network diagram and the haplotype UPGMA diagram revealed the presence of two paternal clades in yaks, indicating two major paternal origins. This finding is consistent with the results of a previous study on the genetic structure of yak sire lineages conducted by [6].

## 5. Conclusions

The current research demonstrated that the SRY region of the Y chromosome in yaks preserves A + T-rich base content and has high paternal population genetic diversity. The Qinghai Plateau, Tianzhu white, and wild yaks were seen to have the highest genetic diversity among the eight studied yak breeds, whereas the Larima yaks had the lowest. Jiuzhi yaks had moderate paternal genetic diversity, comparable to Huanhu (Lake) yaks. Pairwise F_ST comparison revealed low genetic differentiation and high gene flow among Jiuzhi yaks and Qinghai Plateau and Yushu yaks, while extremely high F_ST values with Tianzhu white and wild yaks indicated high genetic divergence and limited gene flow, which may be attributed to geographical or reproductive isolation. Multidimensional scaling (MDS) separated the eight breeds into four genetic groups, in which Jiuzhi yaks were clustered closely with Jinchuan, Yushu, and Huanhu yaks, indicating common patrilineal ancestry. Haplotype network analysis revealed 32 haplotypes, with 12 of them belonging to Jiuzhi yaks and 6 being specific to this population. The occurrence of haplotype H1Y1 in several breeds also corroborates a common origin, whereas the existence of two divergent patrilineal clades in Jiuzhi yaks points to double paternal origins. These results are crucial for understanding Jiuzhi yaks’ paternal genetic framework and evolutionary history and have profound implications for the conservation, genetic management, and selective breeding of native yak populations.

## Figures and Tables

**Table 1:** Specific sample information (Note: Single individual reference sequence used exclusively for phylogenetic inference; not included in statistical tests of diversity).

Community	Sample Size
LARIMA YAK (LRM)	50
Jiuzhi Yak (JZ)	12
Huanhu Yak (HH)	20
Yushu Yak (YS)	7
Jinchuan Yak (JC)	25
Datong Yak (DT)	31
Qinghai Plateau Yak (GY)	1
Tianzhu White Yak (TZ)	1
Wild Yak (WY)	1

**Table 2:** GenBank accession numbers for public SRY sequences incorporated into the dataset.

Assortment	Quantities	Login Number
Wild Yak (WY)	1	XM_005908336
Qinghai Plateau Yak (GY)	1	EU547257
Tianzhu White Yak (TZ)	1	FJ373272

**Table 3:** Base composition of the 690 bp SRY fragment in eight yak populations.

Yak Groups	Base Composition						Segment Length/bp
	A%	C%	G%	T%	A + T%	G + C%	
Qinghai Plateau Yak (GY)	30.00%	20.87%	24.78%	24.35%	54.35%	45.65%	690
Huan hu Yak (HH)	30.13%	20.91%	24.65%	24.30%	54.43%	45.56%	690
Jinchuan Yak (JC)	30.09%	20.89%	24.67%	24.35%	54.44%	45.56%	690
Jiuzhi Yak (JZ)	30.12%	20.88%	24.65%	24.34%	54.46%	45.53%	690
Larima YAK (LRM)	30.15%	20.87%	24.63%	24.34%	54.49%	45.50%	690
Tianzhu White Yak (TZ)	30.16%	20.48%	26.35%	23.02%	53.18%	46.83%	690
Wild Yak (WY)	29.59%	25.95%	23.32%	21.14%	50.73%	49.27%	690
Yushu Yak (YS)	30.00%	20.87%	24.78%	24.35%	54.35%	45.65%	690
on average	30.03%	21.47%	24.73%	23.77%	53.80%	46.19%	690

**Table 4:** Haplotype and nucleotide diversity indices for the SRY fragment. Hd = haplotype diversity; π = nucleotide diversity; MPD = mean pairwise differences.

Yak Population	Sample Size	No. of Haplotypes	Haplotype Gene Diversity	Nucleotide Diversity	Mean Number of Pairwise Differences
Qinghai Plateau Yak (GY)	1	1	1.000 ± 0.000	0.000 ± 0.000	0.000 ± 0.000
Huan hu Yak (HH)	20	10	0.711 ± 0.114	0.003 ± 0.002	1.800 ± 1.084
Jinchuan Yak (JC)	25	12	0.697 ± 0.105	0.002 + 0.001	1.440 ± 0.907
Jiuzhi Yak (JZ)	12	7	0.773 ± 0.128	0.003 ± 0.002	2.000 ± 1.211
Larima YAK (LRM)	50	2	0.040 ± 0.038	0.000 ± 0.000	0.120 ± 0.191
Tianzhu White Yak (TZ)	1	1	1.000 ± 0.000	0.000 ± 0.000	0.000 ± 0.000
Wild Yak (WY)	1	1	1.000 ± 0.000	0.000 ± 0.000	0.000 ± 0.000
Yushu Yak (YS)	7	6	0.952 ± 0.096	0.003 ± 0.002	2.000 ± 1.276
Total/average	117	40	0.772 ± 0.060	0.001 ± 0.001	0.920 ± 0.584

**Table 5:** F_ST values and mean F_ST values among Y chromosome SRY regions of eight yak breeds.

Yak Population	GY	HH	JC	JZ	LRM	TZ	WY	YS
Qinghai Plateau Yak (GY)	0.000							
Huan hu Yak (HH)	0.053	0.000						
Jinchuan Yak (JC)	0.163	−0.001	0.000					
Jiuzhi Yak (JZ)	0.000	0.002	0.008	0.000				
Larima YAK (LRM)	0.887	0.042	0.024	0.115	0.000			
Tianzhu White Yak (TZ)	1.000	0.971	0.977	0.968	0.998	0.000		
Wild Yak (WY)	1.000	0.995	0.996	0.994	1.000	1.000	0.000	
Yushu Yak (YS)	0.000	0.005	0.019	0.000	0.266	0.968	0.994	0.000
Average F_ST	0.915	0.901	0.903	0.900	0.914	0.915	0.915	0.901

**Table 6:** Analysis of molecular variance (AMOVA) for SRY haplotypes. Variance components are partitioned among and within populations; F_ST = 0.907 × 10^−6^ *** (*p* < 0.0001).

Source of Variation	d.f.	Sum of Squares	Variance Components	Percentage of Variation	Fixed Fixation Index
Among populations	7	417.868	4.86381 Va	90.71	
Within populations	109	54.32	0.49835 Vb	9.29	
Total	116	472.188	5.36215		F_ST = 0.9070 × 10^−6^ ***

## Data Availability

No new data were generated in this study. All information supporting the findings is fully contained within the manuscript itself.

## Supplementary Materials

The following supporting information can be downloaded at https://www.mdpi.com/article/10.3390/ani15192783/s1: Table S1: List of main laboratory instruments used in this study, including manufacturer and model specifications for PCR amplification, electrophoresis, sequencing, DNA quantification, and sample storage, ensuring reproducibility of experimental procedures.
