# Supplementary material for: Paternal Lineage and Genetic Diversity of Jiuzhi Yaks Revealed by Y-Chromosome SRY Sequencing
Source: Animals (Basel). 2025 Sep 24;15(19):2783. doi: 10.3390/ani15192783 (PMC12523302; doi:10.3390/ani15192783)
Supplement: Supplementary file 1 [file animals-15-02783-s001.zip › animals-3857473-supplementary.pdf]

Supplementary Table 1 List of main laboratory instruments used in this study, including manufacturer and model specifications for PCR amplification, electrophoresis, sequencing, DNA quantification, and sample storage, ensuring reproducibility of experimental procedures.

name (of a thing)	producer	model number
PCR instrument	ABI, USA	Verity 96well
gel imager	Shanghai Fuzzy Technology Co.	FR-980A
sequencer	ABI, USA	3730XL
refrigerated centrifuge	Anhui Zhongke Zhongjia Scientific Instrument Co.	HC-2518R
Benchtop high speed centrifuge	Hunan Xiangyi Experimental Instrument Development Co.	TD5A-WS
electrophoresis	Beijing Liuyi Instrument Factory	DYY-6C
electrophoresis tank	Beijing Liuyi Instrument Factory	DYCP-32B
Micro Vortex Mixer	Shanghai Husi Analytical Instrument Factory Co.	WH-3
Digital display constant temperature water bath	Taicang Science and Education Equipment Factory	HS-800D
Clean bench	Jiangsu Sujie Clean Chemical Equipment Factory	SW-CJ-1D
BP Series Precision Single Channel Adjustable Pipettes	BBI Canada	F619703 -05
UV-Vis Spectrophotometer	Merinton	SMA4000
freezer cabinet	Qingdao Haier Co.	BCD-256KT
